# Role and Regulation of Transcription Factors in Osteoclastogenesis

**DOI:** 10.3390/ijms242216175

**Published:** 2023-11-10

**Authors:** Tao Jiang, Tianshuang Xia, Fangliang Qiao, Nani Wang, Yiping Jiang, Hailiang Xin

**Affiliations:** 1 School of Pharmacy, Naval Medical University, Shanghai 200433, China; 18756029350@163.com (T.J.); 18305183911@163.com (T.X.); q1956714565@163.com (F.Q.); 2 School of Pharmacy, Fujian University of Traditional Chinese Medicine, Fuzhou 350122, China; 3 Department of Medicine, Zhejiang Academy of Traditional Chinese Medicine, Hangzhou 310007, China; wnn8511@163.com

**Keywords:** osteoclastogenesis, transcription factor, osteoclast, osteoclast differentiation, epigenetics

## Abstract

Bones serve mechanical and defensive functions, as well as regulating the balance of calcium ions and housing bone marrow.. The qualities of bones do not remain constant. Instead, they fluctuate throughout life, with functions increasing in some situations while deteriorating in others. The synchronization of osteoblast-mediated bone formation and osteoclast-mediated bone resorption is critical for maintaining bone mass and microstructure integrity in a steady state. This equilibrium, however, can be disrupted by a variety of bone pathologies. Excessive osteoclast differentiation can result in osteoporosis, Paget’s disease, osteolytic bone metastases, and rheumatoid arthritis, all of which can adversely affect people’s health. Osteoclast differentiation is regulated by transcription factors NFATc1, MITF, C/EBPα, PU.1, NF-κB, and c-Fos. The transcriptional activity of osteoclasts is largely influenced by developmental and environmental signals with the involvement of co-factors, RNAs, epigenetics, systemic factors, and the microenvironment. In this paper, we review these themes in regard to transcriptional regulation in osteoclastogenesis.

## 1. Introduction

Bone is not just a solid physical support structure, but a dynamic, constantly remodeling tissue, which serves as a keeper for marrow cells, a location of blood cell synthesis, and an organ for the regulation of calcium ion homeostasis and endocrine [1,2,3]. Bone remodeling is an essential physiological process that can be affected by a number of variables, including menopause-related hormonal changes, age-related factors, changes in dietary intake, nutritional status, physical activity, medicines, and secondary illnesses [4,5]. Bone remodeling is a lifelong process that results in the formation of a mature, dynamic bone structure by balancing bone formation and bone resorption, a process that involves four main types of bone cells: osteoblasts, osteocytes, bone lining cells, and osteoclasts [4,6]. Osteoblasts are the key bone-producing cells in vertebrates and are primarily responsible for bone formation and bone remodeling [7]. Osteocytes are cells embedded in bones that can control osteoblast and osteoclast activity by regulating local calcium abundance in mineralization and secreting important regulators [8,9]. In adults, bone lining cells are the primary source of osteoblasts and contribute considerably to bone growth following osteoblast ablation [10].

Osteoclasts, as important cells involved in regulating bone metabolism, are mainly responsible for coordinating bone resorption with bone formation to regulate bone remodeling [11]. Regulation of osteoclast differentiation, function, and apoptosis are reliable therapeutic strategies to cope with bone remodeling disorders. Currently, clinical drugs that act on osteoclasts to treat bone-related diseases can be divided into anti-resorptive agents and anabolic agents (Table 1). Anti-resorptive agents include receptor activator of the nuclear factor-kappa B (NF-κB) ligand (RANKL) antibody (Denosumab), calcitonin, and cathepsin K inhibitors (Odanacatib), and anabolic agents that regulate osteoclast differentiation include strontium ranelate. Anabolic agents have greater anti-fracture efficacy than anti-resorptive agents and produce greater functions in enhancing bone mineral density. However, its effects are short-lived, so transition to anti-resorptive agents is required [12]. Denosumab, a human IgG2 monoclonal antibody against RANKL, effectively inhibits osteoclast differentiation and function [13]. However, the anti-resorptive effect is significantly diminished from 7 months after the last injection, which may contribute to the incidence of rebound vertebral fractures [12]. Odanacatib is a specific inhibitor of the osteoclast protease cathepsin K, which prevents osteoclast activity by inhibiting late osteoclast differentiation without affecting normal bone remodeling [14]. However, it leads to an increased risk of cardiovascular events, especially stroke [15]. Bisphosphonates are the most commonly used anti-resorptive drugs. Nevertheless, a growing number of studies indicate that they may cause atypical femur fractures, osteonecrosis of the jaw, gastrointestinal and renal complications, and osteochondrosis. Strontium causes a decrease in bone resorption by inhibiting osteoclast formation and differentiation and promoting their apoptosis [16], which may be related to its inhibition of RANKL expression [17]. However, due to the increased risk of heart problems, the use of strontium has been limited to severe osteoporosis, for which other treatments are not available. In order to decrease the incidence of side effects, further research is required on medications that modify osteoclasts to treat bone remodeling diseases. To develop new strategies for treating bone disease, we need to understand how osteoclasts operate, including the mechanisms that regulate their differentiation. Osteoclast differentiation is regulated by a variety of factors, of which transcription factors are critical. In addition, the action of osteoclast transcription factors can be affected by intercellular communication. Thus, it’s important to understand the molecular mechanisms that regulate osteoclast differentiation and activity, which will provide insight to how hyper-active osteoclasts lead to bone diseases such as osteoporosis. In this review, we summarize the processes involved in the regulation of osteoclast development by osteoclast transcription factors, as well as the variables influencing transcription factor activity, in order to identify a more appropriate strategy to manage bone remodeling by regulating osteoclast differentiation.

## 2. The Origins of Osteoclasts

In the 1970s, studies provided convincing evidence that osteoclasts arise from hematopoietic cells [24,25]. In 1986, osteoclasts were first generated in vitro from highly purified hematopoietic stem cells [26]. In recent years, the advent of emerging technologies such as in vivo cell lineage technologies and single-cell genomics have transformed our understanding of osteoclast biology. A recent study has identified an erythromyeloid progenitor (EMP) that generates long-lasting osteoclast precursors and can contribute to postnatal bone remodeling in both physiological and pathological settings [27]. Moreover, macrophages of EMP origin in the yolk sac generated neonatal osteoclasts capable of creating a niche for hematopoiesis in the postnatal bone marrow [27]. Based on single-cell RNA-sequencing data, the hematopoietic stem cell (HSC) and EMP lineages are two different lineages, and fate-tracking data from the EMP and HSC lineages point to the possibility of cell–cell fusion between these two lineages [27].

### 2.1. EMP-Derived Osteoclasts

EMP-derived monocytes/macrophages mainly produce embryonic osteoclasts, whose role is gradually replaced later. Early EMPs start to generate on embryonic day (E) 7-7.5, which can develop into colony-stimulating factor 1 receptor (CSF1R) + yolk sac macrophages at E8.5, and give birth to a population of tissue-resident macrophages, such as brain microglia [28,29]. Around E8.5, the yolk sac begins to form EMPs, which move and colonize the developing fetal liver by E10.5. After E16.5, they produce macrophages, and go on to dwell in the liver, lungs, brain, and other embryonic organs [30]. Around E8.25–E9, late EMPs arise in the yolk sac and move to the fetal liver to give rise to fetal liver monocytes [29]. These macrophages are kept in the yolk sac in mouse embryos with poor blood flow, indicating that they need blood flow to travel to other embryonic tissues [28,31]. Recent research has demonstrated the importance of EMP-derived embryonic osteoclasts for ensuring teeth eruption, appropriate skull development, and ideal long bone production [32]. Genetic lineage tracing studies have also demonstrated that EMP-derived osteoclasts have a long lifespan of at least six months after birth. EMP-derived osteoclast precursors can move through the bloodstream to the site of bone damage and mature into osteoclasts that are in charge of steady-state bone remodeling and fracture healing [27]. In tissues and organs (especially the brain, liver, and epidermis) of 1-year-old mice, HSC-derived tissue-resident macrophages replaced yolk sac-derived macrophages to some extent. Meanwhile, EMP-derived osteoclast precursor cells were gradually replaced by HSC-derived monocyte progenitors as well [30,32,33]. Additionally, certain osteoclasts produced from long-lived EMP can fuse with monocyte progenitor cells, sustaining the population and functions into maturity [32].

### 2.2. HSC-Derived Osteoclasts

As the foundation of the adult hematopoietic system, HSCs play a key role in the long-term maintenance and generation of all mature blood cell lineages throughout the lifespan of an organism. The hemogenic endothelium, which goes through endothelial-to-hematopoietic transition, is the same source of HSCs as it is for EMPs [34]. Despite sharing the same biological basis, the regulation of HSC development is very different from how EMP emerges [35]. Fetal HSCs emerge at E10.5 in the aorta–gonad–mesonephros (AGM) region, as well as in vitelline and umbilical arteries [36]. They also migrate to the fetal liver, where they give rise to osteoclast precursors [29]. By E16.5, the HSCs migrate to the growing fetal bone marrow, colonize it, and remain into adulthood, where they produce all necessary blood cell lineages [37,38]. Ultimately, HSCs play a critical role in maintaining the hematopoietic system throughout the life of an organism, and bone marrow HSCs establish circulating monocyte-derived macrophages. Fetal monocyte-derived osteoclast progenitors can be generated by fetal HSCs. After birth, bone marrow HSCs continuously differentiate into monocytes/macrophages and dendritic cells, which give birth to osteoclasts [39].

## 3. Osteoclast Differentiation and Maturation

Before generating multinucleated osteoclasts with the potential to absorb bone, HSC- or EMP-derived precursor cells must undergo migration, fusion, and differentiation. The presence of monocytes in the bone marrow cavity is close to the collagen fibers and vascular network [29]. Subsequently, monocytes move to bone tissue after being released into the bloodstream, where they are converted into osteoclast precursor cells (in other terms, bone tissue macrophages) [40]. In addition to the migration mentioned above, osteoclast precursor cells require manager recognition, intercellular adhesion, and membrane fusion [41]. Membrane fusion of osteoclasts is not random but has a tightly controlled mechanism [42]. The basis for this selection is intercellular heterogeneity, which includes nuclearity, mobility, and maturity [41,42,43]. The formation of this heterogeneity also involves osteoclast fusion factor, CD47, DC-specific transmembrane protein (DC-STAMP), and syncytin-1 [41]. Mononuclear osteoclast precursors move and adhere to each other before membrane fusion, a process that depends on alphav beta3 (αvβ3) integrin localized to podosomes at the leading edge of osteoclasts [44]. DC-STAMP and osteoclast stimulatory transmembrane protein (OC-STAMP) are “master fusogens” during osteoclast multinucleation. The regulation of DC-STAMP is tissue-specific, and its overexpression leads to osteoclast hyper-multinucleation and exhibits an osteoporotic phenotype [45,46]. The protein structure of OC-STAMP is similar, but not identical to that of DC-STAMP [46]. Under RANKL stimulation, OC-STAMP-deficient bone marrow cells were able to develop into TRAP-positive osteoclasts but unable to fuse into multinucleated cells [47]. Additionally, it has been suggested that the tetraspanins (CD9, CD81), CD44, CD47, syncytin-1, PIN1 (peptidyl-prolyl cis-trans isomerase NIMA-interacting 1), and CD44 are essential for osteoclast fusion and multinucleation [48,49,50,51,52].

**Figure 1:**
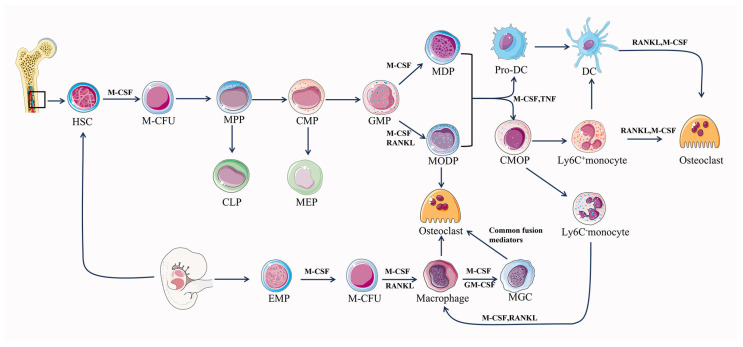
Differentiation pathway of osteoclastogenesis. M-CFU, myeloid colony-forming units; MPPs, multipotent progenitors; CMPs, common myeloid progenitors; CLPs, common lymphoid progenitors; MEPs, megakaryocyte/erythrocyte progenitors; MODPs, macrophage/osteoclast/dendritic cell progenitors.

The differentiation of osteoclasts is primarily dependent on a tumor necrosis factor (TNF) family, RANK, RANKL, osteoprotegerin (OPG), and M-CSF [53,54]. Hematopoietic stem cell bone marrow colony-forming units (M-CFUs), generated from HSCs or EMPs, are the main source of myeloid cell generation [55,56] (Figure 1). Early PU.1 and MITF expression in M-CFUs causes the emergence of M-CSFR (the receptor for M-CSF) [56]. Furthermore, HSC-derived M-CFUs can develop into MODPs in the presence of M-CSF and RANKL. But in the absence of M-CSF, HSC-derived M-CFUs can differentiate into M-CSF-dependent macrophages and dendritic cell progenitors (MDPs) [56]. Subsequently, in the presence of M-CSF, MDP can differentiate into dendritic cells, and in the further presence of M-CSF as well as TNF, MDP can differentiate into monocytes [56]. Common monocyte progenitors (cMoPs) are a well-defined transient intermediate in the differentiation of MDPs into monocytes, which differ from MDPs in their lack of CD135 (also known as FLT3) expression and their loss of conventional DC (cDC) or pDC potentials [57,58]. It has been shown that trans-transferred cMoPs can give rise to Ly6C+ and Ly6C- monocyte subpopulations, such as M-CSF and TNF, under appropriate stimulation [57,59]. In response to M-CSF and RANKL stimulation, early-stage Ly-6C+ monocytes show a significant propensity for osteoclast commitment while still having the ability to differentiate into Ly6C- monocytes [56,58]. Ly6C- monocytes and Ly-6C+ monocytes can differentiate directly into osteoclasts under the co-induction of M-CSF and RANKL, and Ly6C-monocytes can differentiate into macrophages under the induction of M-CSF alone. Then, macrophages fuse into osteoclasts under the co-induction of M-CSF and RANKL [56]. When activated by M-CSF or GM-CSF with interleukins (IL-4, IL-13), macrophages can produce multinucleated giant cells (MGCs) in pathogenic circumstances [56]. MGCs continue to develop into osteoclasts when common fusion mediators are present. Additionally, premature DCs can develop into typical DCs, while they can also become osteoclasts when exposed to M-CSF and RANKL [60]. EMP-derived M-CFUs can generate macrophages under the induction of M-CSF [56]. Furthermore, EMP-derived mature osteoclasts can maintain postnatal survival and function mediated by the sequential acquisition of new nuclei from long-lived syncytia in HSC-derived blood leukocytes, rather than by lateral fusion or proliferation of osteoclast precursors to achieve new renewal [32]. Osteoclast precursor cells undergo a series of steps to finally differentiate and form mature osteoclasts, which realize the roles of bone resorption and regulation of bone homeostasis.

## 4. Intercellular Communication in Osteoclastogenesis

The mechanisms of bone development, repair, and regeneration depend on the interaction and communication between the osteoclast and other surrounding cells, while osteoclastogenesis is also regulated to a large extent by other cells. To further explore the regulatory mechanisms of osteoclastogenesis, we investigated the major cells involved in osteoclastogenesis, including osteoblasts, osteocytes, adipocytes, and immune cells.

### 4.1. Osteoclast–Osteoblast Communication

**Figure 2:**
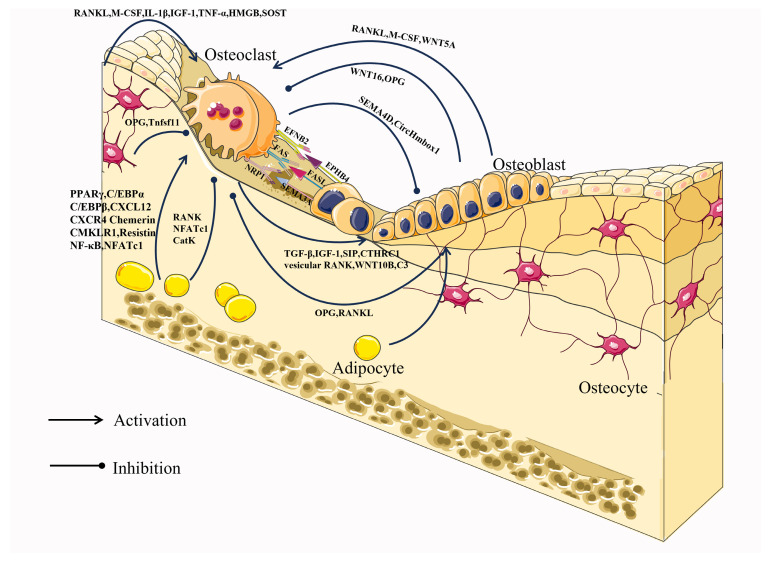
Intercellular communication between osteoclasts and osteoblasts, osteocytes, and adipocytes.

Through direct cell-to-cell contact or secretion of proteins and RNA, osteoblasts and osteoclasts communicate to manage cellular activity, survival, and differentiation. Direct interactions between osteoblasts and osteoclasts regulate the differentiation and survival of osteoblasts or osteoclasts mainly through bidirectional regulation of the EFNB2 (Ephrin B2)-EPHB4, FAS Ligand (FASL)-FAS, or Semaphorin 3A (SEMA3A)-NRP1 signaling pathways [61] (Figure 2).

Activation of the osteoblast surface molecule EPHB4 is initiated by binding to the cell surface molecule Ephrin B2 of osteoblasts and inhibits osteoclast differentiation by blocking osteoclast-generated c-Fos/NFATC1 [62]. In contrast, EFNB2-mediated activation of EPHB4 promoted osteoblast differentiation and inhibited apoptosis [63]. FAS and its ligand FASL constitute the FAS-FASL signaling pathway, and play a central role in the physiological regulation of apoptosis in FAS-expressing cells [64]. Estrogen up-regulates FASL expression in osteoblasts and leads to pro-osteoclast apoptosis [65]. SEMA3A, generated by osteoblast spectrum cells, can also inhibit RANKL-induced osteoclast differentiation by binding to neuropilin-1 (NRP1) and promote osteoblast development through the WNT/-linked protein pathway [62,66].

The cytokines M-CSF and RANKL play a crucial role in the development of osteoclasts and their expression is common among a variety of cell types, including osteoblast lineage cells. They regulate significant aspects of osteoclast formation and operation. M-CSF, a hematopoietic growth factor, is secreted by osteoblasts and bone marrow stromal cells and promotes osteoclastogenesis by binding to its cognate receptor C-FMS on the surface of osteoclasts and monocytes/macrophages [44]. RANKL is abundantly expressed in osteoblasts, osteocytes, activated T lymphocytes, and lymph nodes, and it binds to its cognate receptor RANK on the surface of osteoclasts and osteoclast precursors, causing differentiation, fusion, and activation of osteoclasts [67]. The function of RANKL is not only directly regulated by OPG but also indirectly regulated by WNT5A and WNT16. OPG is a secreted glycoprotein that is synthesized by a variety of cell types, including osteoblasts, and is thought to act as a decoy receptor that binds to RANKL and inhibits the regulation of osteoclast differentiation and activation by obstructing RANKL-RANK interactions [68,69]. Furthermore, WNT5A, released by osteoblasts, also promotes RANKL-induced osteoclastogenesis by activating the Jun-N-terminal kinase (JNK) MAPK pathway, which increases RANK expression in osteoclasts [62]. WNT16, mainly produced by osteoblasts, directly inhibits osteoclastogenesis through the non-canonical JNK MAPK pathway, and WNT16-induced JUN phosphorylation also indirectly inhibits osteoclastogenesis by increasing OPG expression [62].

In addition, osteoclasts can regulate the differentiation and function of osteoblasts. The process of osteoclast-mediated bone resorption is accompanied by the release of TGF-β and IGF-1 from the bone matrix to induce osteoblast-mediated bone formation [70,71]. In addition, osteoclasts can also promote osteoblast differentiation by secreting Sphingosine 1 Phosphate (S1P), Collagen Triple Helix Repeat Containing 1 (CTHRC1), vesicular RANK, WNT10B, and Complement Component C (C3), as well as inhibit osteoblast differentiation by secreting Semaphorin 4D (SEMA4D) [72,73,74,75,76]. Moreover, osteoblasts and osteoclasts can interact through exosomes. Circ_0008542 and miR-503-3p in osteoblast exosomes promote osteoclast-induced bone resorption through m6A methylation and inhibit osteoclast differentiation through down-regulating Hpse gene expression, respectively [77]. Conversely, miR-23a-5p in osteoclast exosomes inhibited osteogenic differentiation by regulating *Runx2* [78]. In addition, TNF-α can reduce osteoblast differentiation through osteoclast exosomes with low expression of CircHmbox1 [79]. As the sole cell for bone resorption and the primary cell for bone formation, intercellular communication between them is essential for bone remodeling. While the intercellular communication between osteoclasts and osteoblasts has been thoroughly studied, there is still room for further investigation into their epigenetic aspects.

### 4.2. Osteoclast–Osteocyte Communication

Osteocytes represent the most fully differentiated cell type within the osteoblast lineage and possess the characteristic stellate morphology that is distinct to these cells. This morphology facilitates physical interaction and exchange of molecules between osteocytes, as well as neighboring cells within the bone surface or bone marrow area [80]. Osteocytes are thought to be the first mechanosensory cells. However, recent research has found that osteocytes produce factors that not only regulate bone cells, but also regulate organs such as the kidneys. The regulatory role of osteocytes on osteoclasts is mainly through the RANKL/OPG mechanism (Figure 2). Osteocytes are the main source of RANKL during osteoclastogenesis, and selective deletion of the gene for RANKL in osteoblast populations (but not osteoclasts or precursors), Tnfsf11, results in defective osteoclastogenesis [81,82,83]. Osteocytes supply their produced RANKL to osteoclast precursors in a membrane-bound form via osteocyte dendritic processes that extend beyond the bone surface and into the bone marrow and periosteal regions [81]. Also, osteocytes are an important source of OPG, which can act as a soluble decoy receptor for RANK and inhibit osteoclast formation. Thus, osteocytes can promote or inhibit the control of osteoclast formation through the expression/supply of RANKL as well as the expression/supply of OPG [83]. In addition, osteocytes can also promote osteoclast differentiation and function by secreting M-CSF, interleukin 6 (IL-6), IL-1β, IGF-1, tumor necrosis factor alpha (TNF-α), and HMGB1 [84,85,86,87,88,89]. Among them, IL-6 and IGF-1 indirectly promote osteoclastogenesis by promoting the expression of osteoblast RANKL alone and M-CSF and RANKL at the same time, which also reflects the close connection between osteoblasts, osteoclasts, and osteocytes. SOST is specifically expressed in osteocytes stimulated by mechanical stress and antagonizes Wnt-induced bone formation by binding to the extracellular domain of osteoblast low-density lipoprotein receptor-related protein 5/6 [90]. Osteocytes also regulate osteoclastogenesis and function through the expression of sclerostin (SOST), which stimulates osteoclastogenesis in a RANKL-dependent manner, as well as regulate osteoclast function by modulating the expression of carbonic anhydrase 2, cathepsin K (CTSK), and TRAP [90].

### 4.3. Osteoclast–Adipocyte Communication

**Figure 3:**
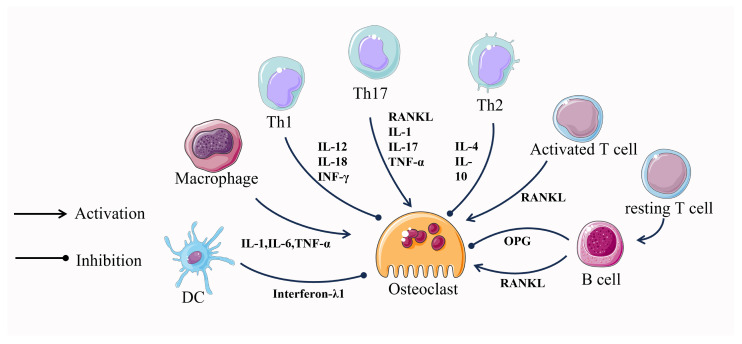
Intercellular communication between osteoclasts and immune cells.

The regulation of osteoclast differentiation is also influenced by adipocytes. In addition to transcription factors such as PPARγ, CCAAT enhancer binding protein alpha (C/EBPα), and C/EBPβ, they achieve this via a variety of adipokines in bone marrow adipose tissue, including visfatin, chelatan, reticulin-1, leptin, and resistin (Figure 3). Among them, visfatin, leptin, and omentin-1 negatively regulate the differentiation process of osteoclasts.

During the differentiation of osteoclasts, significant transcription factors, including PPARγ, C/EBPα, and C/EBPβ, are expressed in hematopoietic lineage cells. Along with RANKL expressed by bone marrow adipocytes, these factors are essential for both osteoclast differentiation and activity. However, they may have additional adverse effects on bone health by altering communication between osteoblasts and osteoclasts, and further promoting osteoclastogenesis and bone resorption [91,92]. PPARγ can also promote osteoclastogenesis by inducing c-Fos through binding to homologous response elements within the c-Fos promoter in HSCs [93]. Furthermore, adipocytes regulate the differentiation of osteoclasts by controlling RANKL and OPG in osteoblasts, in addition to these transcription factors. Visfatin has two aspects of action: promoting osteoclast proliferation and collagen secretion in an insulin receptor-mediated manner and blocking osteoclast differentiation by inhibiting the expression of RANK, NFATc1, and CatK [94,95]. Similarly, the role of Omentin-1 and leptin covers two aspects: promoting osteoclast proliferation and indirectly inhibiting osteoclast differentiation by inducing OPG production by osteoblasts and inhibiting RANKL production [92,96,97].

Furthermore, adipocytes can promote osteoclast differentiation, function, and expression of adhesion-related molecules through the CXCL12/CXCR4 signaling pathway, and Chemerin/CMKLR1 signaling regulates the expression of key osteoclast transcription factor NFATc1 to induce osteoclast differentiation and matrix resorption [98,99]. In addition, Chemerin and Resistin can induce osteoclast differentiation through a signaling mechanism involving NF-κB [100]. The communication between adipocytes and osteoclasts involves a variety of substances. However, the role of these substances in regulating osteoclastogenesis is still inextricably linked to the transcription factors.

### 4.4. Osteoclast–Immune Cell Communication

The close relationship between the immune system and the skeletal system has been under scrutiny for many years. In 2000, Aaron and Choi coined a new term, osteoimmunology, an emerging field of research focused on the interaction between immune cells and the skeletal system [101]. Numerous molecules, including cytokines, chemokines, hormones, receptors, and transcription factors, are shared between the immune and skeletal systems. Immune cells and bone cells communicate with one another both normally and pathologically [102]. It seems that almost all immune cells communicate with bone cells and vice versa, where T cells and their helper cells (Th1, Th2, Treg, and Th17) as well as various other immune cells (B cells, DCs, macrophages, etc.), especially T cells, which are involved in the regulation of the osteoclastogenesis process [103,104] (Figure 3).

Activated T cells induce osteoclastogenesis by directly affecting osteoclast precursor cells through the expression of RANKL [104]. In contrast, resting T cells inhibit osteoclastogenesis through a mechanism that involves mediating the complete suppression of OPG by B cells [104,105]. In addition to this, T cells can also differentiate into CD4 and CD8 T cells which continue to influence osteoclastogenesis. CD4 T cells have no effect on osteoclast formation, whereas depletion of CD8 T cells results in a 40% increase in osteoclast formation [106]. This suggests that differentiated T cells play a role primarily in promoting osteoclastogenesis.

Cytokines produced by T-cell helper cells also play an important role in osteoclastogenesis. IL-12 and IL-18, which induce differentiation of Th1 cells, and INF-γ, a cytokine produced by Th1, together exert an inhibitory effect on osteoclast formation [107,108]. However, Th1 cells can also promote bone resorption by regulating the RANKL/OPG balance through synthetic soluble RANKL and by interfering with the coupling of osteoclasts and osteoblasts through TNFα de-differentiation of osteoblasts [109]. Cytokines produced by Th2, mainly IL-4 and IL-10, exert an inhibitory effect on osteoclast formation [108,110]. CTLA-4, expressed by Tregs, binds to CD80/CD86 and inhibits T cell activation, thereby suppressing T cell-induced RANKL expression and inhibiting osteoclastogenesis [111]. Th17 cells can directly influence osteoclastogenesis through the expression of RANKL. In addition, Th17 cells can indirectly regulate osteoclastogenesis by secreting IL-17, leading to the secretion of TNF-α and IL-1, which in turn induce the expression of RANKL in cells, supporting osteoclastogenesis [112].

During in vitro culture, B cells were induced by M-CSF and RANKL to express RANKL and differentiate into osteoclasts, while RANKL expression on B cells was increased in severe periodontitis [113,114]. In the presence of M-CSF and RANKL, DCs can transdifferentiate into osteoclasts in vitro and in vivo and are directly involved in osteoclastogenesis. However, DC-derived interferon-λ1(IFN-λ1) inhibits osteoclastogenesis [115]. Like DCs, macrophages in the presence of M-CSF and RANKL can also differentiate into osteoclasts. Macrophage production of pro-inflammatory cytokines, such as TNF-α, IL-1, and IL-6, directly promotes RANKL-induced osteoclast differentiation [114]. After years of research, the mechanisms of regulation of osteoclastogenesis by immune cells are becoming clear, and this interdisciplinary approach may lead to the development of targeted therapies.

In osteoclastogenesis, intercellular communication between osteoclasts and osteoblasts involves the transcription factors c-Fos and NFATC1, as well as M-CSF and RANKL, which regulate transcription factors. Furthermore, the MAPK pathway, which regulates the expression of transcription factors, is involved. Intercellular communication between osteoclasts and osteocytes also involves M-CSF and RANKL. During osteoclastogenesis, intercellular communication between osteoclasts and adipocytes involves the important transcription factors C/EBPα, c-Fos, NFATc1, and NF-κB, as well as RANKL, which regulates transcription factors. Intercellular communication between osteoclasts and immune cells is likewise inextricably linked to RANKL. RANKL and M-CSF are key factors that regulate osteoclasts and can modulate osteoclastogenesis by further regulating the expression of transcription factors. As a result, cellular interactions and communication in osteoclastogenesis are closely linked to the expression of osteoclast transcription factors. Therefore, we conducted further studies on the major transcription factors of osteoclasts.

## 5. Transcriptional Regulation in Osteoclastogenesis

Osteoclast transcription factors are essential for the osteoclastogenesis process, bone remodeling, and the establishment and maintenance of bone homeostasis. It may offer novel therapeutic possibilities for the treatment of bone-related illnesses by digging into the mechanisms of these transcription factors and the regulatory variables.

### 5.1. NFATc1

Nuclear factor of activated T cell cytoplasm (NFATc), a family of transcription factors originally identified in T cells, has five members (NFATc1 to NFATc5) that function in the immune system and beyond [116]. NFATc1, a widely expressed member of the NFAT family, functions in endothelial cells of the endocardial cushion (ECC), skeletal muscle fibers, T lymphocytes, osteoblasts, and osteoclasts [117,118,119,120].

RANKL-RANK signaling initiates osteoclastogenesis by activating phospholipase C2 (PLC2), which generates inositol-1,4,5-trisphosphate (IP3) from the plasma membrane phospholipid phosphatidylinositol 4,5-bisphosphate [PI (4,5) P2]. IP3 then induces calcium oscillations, which are essential for NFATc1 activation [121]. In the early stages, RANKL binds to RANK and TNF receptor-associated factor 6 (TRAF6), which leads to the activation of downstream molecules such as NF-κB, c-Jun, and P38 [66]. The initial induction of NFATc1 is triggered by the collaboration of NF-κB and NFATc2 [116,122]. The induction of c-Fos and NFATc1 can be mediated by the P38 signaling pathway [123]. The above molecules work together to activate NFATc1 in a positive manner. Additionally, NFATc1 has the ability to bind to its own promoter, strongly inducing NFATc1 [116]. The co-stimulatory signal produces Ca2+ oscillation via activated phospholipase Cγ2 (PLCγ2) in conjunction with c-Fos/AP-1 in the intermediate stage of signaling, where Ca2+ signaling enhances the robust synthesis of NFATc1 [124]. NFATc1 translocates into the nucleus during late osteoclastogenesis, where it promotes a slew of osteoclast-specific target genes involved in cell fusion and function [124]. In particular, osteoclast precursors that are ectopically expressed with NFATc1 undergo differentiation into mature cells in the absence of RANKL. NFATc1-deficient embryonic stem cells are unable to differentiate into osteoclasts, and targeted disruption of NFATc1 in mouse hematopoietic cells results in increased bone mass with a marked reduction in osteoclasts [125,126]. NFATc1 is identified by transcriptomic profiling as a master regulator of the osteoclast transcriptome, enhancing the expression of multiple genes required for bone resorption. Osteoprotegerin is a decoy receptor for RANKL, previously thought to be an inhibitor of osteoclast-derived bone resorption, and its expression in osteoclast progenitors can be directly inhibited by NFATc1 [126]. NFATc1 also has a positive regulatory effect on DC-STAMP in conjunction with c-Fos. The deficiency of NFATc1 and c-Fos leads to impaired osteoclast fusion and significantly reduced bone resorption activity [127]. The role of NFATc1 is also regulated by many other factors. The phosphorylation of many NFAT kinases, including casein kinase 1 (CK1), glycogen synthase kinase 3 (GSK3), and dual specific tyrosine phosphorylation-regulated kinases (DYRKs) can all regulate NFATc1 function [128]. The transcription factor signal transducer and activator of transcription 3 (STAT3) are essential for cell survival and function. STAT3 deficiency is associated with decreased NFATc1 expression, decreased osteoclast differentiation, and increased bone mass. Furthermore, STAT3 can activate NFATc1 transcription by interacting with its promoter [129]. IRFs, or interferon regulatory factors, are involved in a number of biological activities, such as cytokine signaling, controlling cell proliferation, and hematopoietic development [130]. IRF2 in osteoclast precursor cells affects osteoclast differentiation by regulating the RANKL-induced NF-κB/NFATc1 signaling pathway [130]. Kruppel-like factor 2 (KLF2) is thought to have a role in controlling how different types of cells differentiate, proliferate, and survive. Overexpression of KLF2 in osteoclast precursor cells inhibits osteoclast differentiation by down-regulating c-Fos, NFATc1, and TRAP expression [131]. The function of KLF2 in the regulation of osteoclastogenesis is linked to the activation of NF-κB transcription through the control of p65. This function is positively regulated by interferon regulatory protein 2 binding protein 2 (IRF2BP2). Uremic toxins, such as indole sulfate (IS), affect NFATc1 expression via regulating aryl hydrocarbon receptor (AhR) signaling in osteoclastogenesis [132].

In conclusion, NFATc1 appears to be a prime target for anti-osteoclast therapy because it is implicated in every stage of osteoclast production and activation. Thus, to better understand the molecular mechanisms behind NFATc1 regulation in osteoclasts, novel therapeutic strategies for bone illnesses defined by an excess of osteoclast production and activity may be developed.

### 5.2. MITF

MITF is recognized to have a role in melanocyte development, and mounting research supports its significance in aging and senescence, notably in melanocyte senescence. MITF primarily controls senescence-associated secretory phenotype (SASP) synthesis, oxidative stress, DNA repair, cell cycle, and melanocyte senescence [133]. Like all transcription factors, MITF binds to certain DNA sequences and controls the expression of its target genes by up- or down-regulating them. The vast array of biological processes that MITF appears to coordinate sets it apart from its contemporaries. These include cell survival, differentiation, proliferation, invasion, senescence, metabolism, and DNA damage repair [134]. The primary function of MITF in osteoclasts is to control osteoclast differentiation, which is connected to RANKL [135]. It has been discovered that the promoter activity of CTSK can be independently stimulated by activator protein 1 (AP-1), MITF, and NFATc1. Any two variables can be combined to further boost CTSK promoter activity. However, the most significant increase is caused by the combination of AP-1 (c-Fos/c-jun) and NFATc1 [136]. Although MITF was previously found to act upstream of NFATc1, a study revealed that NFATc1 was necessary for the induction of MITF isoform-E (MITF-E), and MITF expression had minimal impact on it. During osteoclastogenesis, MITF-E serves as a tissue-specific modulator for activities that follow NFATc1 activation [137]. Additionally, NF-κB increased MITF expression by down-regulating miR-1276 to promote osteoclast differentiation [138]. In osteoclasts, MITF is primarily responsible for regulating osteoclast differentiation, which is linked to RANKL and CTSK, and controlled by NFATc1 and NF-κB.

### 5.3. C/EBPα

In osteoclast precursors and osteoclasts, C/EBPα expression was high, and the combined presence of M-CSF and RANKL could significantly induce high C/EBPα expression [139]. Two important OC transcription factors, c-Fos and C/EBPα, are up-regulated when the RANKL binds to its receptor RANK on osteoclast precursors [140]. Osteoclast precursors are capable of initiating osteoclastogenesis by overexpressing c-Fos or C/EBPα, resulting in up-regulation of osteoclast genes in the absence of RANKL. The overexpression of C/EBPα promotes OC development more potently than that of c-Fos, despite the fact that c-Fos fail to up-regulate C/EBPα in osteoclast precursors [140]. Deficiency of the bone marrow master regulator C/EBPα in osteoclasts leads to impaired terminal differentiation, activation, and function. Specifically, it can lead to a mild reduction in osteoclast numbers, impaired osteoclast polarization, and impaired actin formation and bone resorption, finally completely blocking ovariectomy-induced bone loss. However, it does not affect bone formation and monocyte/macrophage development [141]. C/EBPα deficiency decreases the expression of osteoclast functional genes such as Ctsk, Atp6i (Tcirg1), and osteoclast regulatory genes such as nuclear factors expressing c-Fos [141,142]. However, while PU.1 up-regulated C/EBPα, C/EBPα could not up-regulate PU.1 [143]. Impaired osteoclastogenesis in bone marrow cells of C/EBPα-deficient mice is rescued by overexpression of c-Fos, which is directly up-regulated by C/EBPα [139]. Furthermore, the crucial cis-regulatory element (CCRE) is found in the promoter region of NFATc1 and is intimately related to C/EBPα [141]. Overall, C/EBPα is expected to be a promising new target for the treatment of osteolytic diseases.

### 5.4. PU.1

The Sfpi1 (Spi-1) gene encodes PU box-binding protein (PU.1), which produces a protein with 272 amino acids (predicted MW of 31 kDa) connected to the Spi-B and Spi-C Ets-family factors (both share around 40% amino acid similarity) [144]. The transcription factor PU.1, an important member of the E-twenty-six (ETS) family, plays an important role in immune cell differentiation and is required for the formation of practically all blood cell lineages [145,146]. The absence of PU.1 in osteoclasts caused severe osteopetrosis [147]. PU.1 plays a crucial role in promoting changes in DNA methylation during osteoclast differentiation [148]. PU.1 expression is increased during RANKL-induced osteoclast differentiation, and it’s proportional to the rate of macrophage differentiation to osteoclasts, and its deletion influences osteoclast differentiation [149,150]. PU.1 promotes osteoclast development by directly binding to the NFATc1 promoter and transactivating NFATc1 expression [151]. Overall, PU.1 can promote osteoclast development by promoting DNA methylation and transactivating NFATc1 expression.

### 5.5. NF-κB

Nuclear factor-κB (NF-κB) is a transcription factor involved in signaling pathways essential for normal cell function and development. Osteoclast differentiation requires NF-κB activation. NF-κB consists of five transcription factors: p50/p105, p52/p100, p65 (relA), c-Rel, and RelB [152]. It has been discovered that NF-κB1 (p50) and NF-κB2 (p52) double knockout mice exhibit severe osteopetrosis due to a lack of osteoclasts [152]. This suggests that NF-κB can directly control osteoclast differentiation, and recent studies showed that NF-κB could also indirectly control osteoclast differentiation. However, impaired osteoclastogenesis caused by NF-κBp50/p52 or c-Fos deletion can be rescued by overexpression of NFATc1 [153]. Moreover, the deficiency of p65 results in the decrease of osteoclastogenesis and this deficiency is sensitive to apoptosis [154]. RelB regulates osteoclast differentiation in both directions. When the RelB protein is up-regulated by tumor necrosis factor α, it promotes osteoclast formation capacity. However, elevated RelB also inhibits terminal differentiation of osteoclasts by suppressing NFATc1 expression [154]. The regulation of osteogenesis by NF-κB is impacted similarly by RANKL. The removal of RANKL or RANK and the suppression of RANKL-RANK signaling can all reduce NF-κB activity and impede osteoclastogenesis. Nevertheless, embryonic lethality has been reported in mice involved in NF-κB signaling [155]. Hence, it is essential to take into account the potential of causing severe side effects simply by inhibiting NF-κB.

### 5.6. c-Fos

Proteins from the Activator Protein-1 (AP-1) family, especially in the Fos proteins, play a significant role in osteoclast regulation [156]. The expression level of Fos positively correlated with the differentiation ability of osteoclasts and improved the inhibition of osteoclast differentiation [157,158]. Additionally, co-transfection of several jun family members with c-Fos can greatly boost the expression of CTSK promoters [158]. The AP-1 complex, which contains c-Fos, is essential for the auto-amplification of NFATc1, which allows for the robust induction of NFATc1 [159]. Meanwhile, transcriptional repression of c-Fos decreases the expression of NFATc1. Furthermore, through the activation of the autophagy, PI3K/Akt, and TAK1/S6 signaling pathways, sustained activation of c-Fos can undo the OPG-mediated inhibition of osteoclastogenesis [160]. RANKL signaling induced by activated T cells is a central link in the signaling network leading to osteoclast-mediated bone loss, with subsequent activation of the key transcription factors Fos/AP-1, NF-κB, and NFATc1 playing a critical role [161]. The function of c-Fos in osteoclastogenesis involves the promotion of NFATc1 and the inhibition of OPG. This process is regulated by autophagy, RANKL, PI3K/Akt, and TAK1/S6 signaling.

### 5.7. DAP12, TREM2, and FcRγ

Immunoreceptor tyrosine-based activation motif (ITAM) adaptor–receptor complexes elicit costimulatory signals for osteoclastogenesis [162]. Thus, DNAX-activating protein 12 kD (DAP12) and Fc receptor γ chain (FcRγ), as adaptors of ITAM, are essential for the process of osteoclastogenesis. TREM2 (Trigger Receptor Expressed on Myeloid Cells-2) is the main receptor associated with DAP12 in osteoclasts. Lack of DAP12 or TREM2 results in the blockage of the osteoclastogenesis process and the formation of mononuclear osteoclasts [163]. Linkage of DAP12 to TREM2 promotes osteoclast differentiation and function by activating phosphatidylinositol 3-kinase (PI3K), inducing intracellular calcium (Ca^2+^) mobilization, and promoting actin reorganization [164]. In this process, DAP10 can promote TREM2- and DAP12-dependent recruitment of PI3K to the signaling complex. In contrast, Src homology 2 (SH2) structural domain-containing inositol phosphatase-1 (SHIP1) binds to DAP12 in a SH2 structural domain-dependent manner, preventing PI3K from being recruited to DAP12, thus inhibiting TREM2- and DAP12-induced signal transduction [164]. As with DAP12, the deletion of FcRγ results in severe defects in osteoclastogenesis [165]. In the absence of DAP12, FcRγ can normalize the DAP 12-deficient skeleton via αvβ3 [166]. DAP12, TREM2, and FcRγ are tightly linked and play important regulatory roles in osteoclastogenesis. As research progresses, more complexity is being revealed about the regulation of DAP12, TREM2, FcRγ, and ITAM signaling, but these complex regulatory mechanisms are important for the regulation of bone remodeling.

### 5.8. Other Transcription Factors Regulating Osteoclast Differentiation

#### 5.8.1. PLC-Gamma

Members of the phospholipase Cγ(PLCγ) family, PLCγ1 and PLCγ2, are important regulators of signaling pathways downstream of growth factor receptors, integrins and immune complexes and play an important role in osteoclastogenesis. PLCγ1 and PLCγ2 play important roles in osteoclast differentiation mainly by regulating DAG-mediated calcium oscillations and up-regulation of the transcription factor NFATc1 [167,168]. Differently from PLCγ2, PLCγ1 shRNA also inhibits osteoclast differentiation by suppressing CSF-1-dependent proliferation and β-catenin/cyclinD1 levels [168]. In addition, the ability of PLCγ2 to regulate NFATc1 expression was positively regulated by JNK/NF-κB, whereas the ability of PLCγ1 to regulate NFATc1 expression was positively regulated by TLR4/TRAF6 [169]. Collectively, PLCγ1 and PLCγ2 are involved in osteoclastogenesis by regulating the expression of the key transcription factor NFATc1.

#### 5.8.2. AKT

Akt is a serine/threonine kinase that is a central node in many signaling pathways that are activated by a variety of growth signals and regulate the function of many downstream proteins involved in cell survival, proliferation, migration, and metabolism. Akt can regulate osteoclastogenesis through multiple pathways. Activated AKT promotes osteoclastogenesis by improving nuclear translocation of NFATc1, promoting c-Fos expression, and increasing the expression of downstream genes including CTSK [170,171,172]. The role of AKT in promoting NFTAC1 expression can be achieved by inhibiting GSK3β expression and promoting mTOR expression. Meanwhile, RANKL can positively regulate AKT expression by modulating the expression of PI3Kδ [171]. In conclusion, Akt is involved in osteoclastogenesis mainly through the regulation of NFTAC1 and is regulated by RANKL.

#### 5.8.3. Src

The tyrosine kinase Src is a proto-oncogene that is commonly expressed in various tissues and is involved in cell growth, migration, and attachment, as well as being one of the molecules that regulate the homeostasis of bone metabolism. Src promotes osteoclast bone resorption activity by facilitating the formation of actin rings, while inhibiting osteoclast function through the inhibition of RUNX2 [173]. Activation of Src is achieved by RANKL, M-CSF, and integrin signaling via PTP-oc [174]. Deletion of Src results in attenuated tyrosine phosphorylation of Tks5, leading to impaired cell fusion during the process of osteoclastogenesis [175]. At present, the regulation of osteoclasts via src mainly focuses on the regulation of osteoclast function, and the role of src on osteoclastogenesis needs further in-depth study.

#### 5.8.4. RIP140

Expressed in a widespread manner, the transcription cofactor receptor-interacting protein 140 (RIP140) regulates a number of biological processes, including metabolism, inflammatory response, and cell division [176,177,178]. RIP140 expression in osteoclast precursors has been shown to be essential for osteoclast development, activity, and coupled bone production in earlier investigations. The role of RIP140 in estrogen-mediated osteoclastogenesis and bone resorption may be crucial. Osteoclasts produce RIP140 much more when estrogen is present, and this increased expression of RIP140 via the Fas/FasL pathway has the potential to cause osteoclast death [179]. The inhibition of osteoclast production and bone resorption activity by estrogen might be greatly reduced by the reduction of RIP140 [179]. Mechanistic analysis showed that RIP140 inhibited osteoclast differentiation mainly by suppressing the formation of a transcriptional repressor complex between osteoclast genes and testis receptor 4 [180]. Reducing osteoclastogenesis by blocking the degradation of RIP140 protein may be a new strategy for treating bone loss.

#### 5.8.5. TFE3

Metabolic genes are controlled by an EBox found in their promoters, and transcription factor for immunoglobulin heavy-chain enhancer 3 (Tfe3) acts as a transactivator of these genes [181]. It is involved in pathological processes like translocations that underlie several cancer disorders, as well as physiological processes like osteoclast and macrophage development [181]. The tumor suppressor gene follicular protein (Flcn) in osteoclast precursors can regulate oxidative phosphorylation and purine metabolism by inhibiting the nuclear localization of the transcription factor Tfe3. This suppresses the expression of the target gene Pgc1, effectively impeding the acceleration of osteoclastogenesis [182].

#### 5.8.6. BCL-6 and Blimp1

There is a novel osteoclastogenesis regulatory pathway involving two transcriptional repressors [B-cell lymphoma 6 (Bcl6) and B-lymphocyte-inducible maturation protein 1 (Blimp1)] in response to RANCL stimulation [127]. Bcl6 inhibits the expression of osteoclast genes (e.g., DC-STAMP, NFATc1, and Ctsk) and osteoclast differentiation. However, the inhibitory effect of Bcl6 is negatively regulated by Blimp1 [127]. By modulating osteoclast generation, the Blimp1-Bcl6-osteoclast molecular axis can crucially govern bone homeostasis and may provide the molecular basis for innovative therapeutic techniques [183].

#### 5.8.7. DDIT3

The C/EBP family member DNA damage inducible transcript 3 (DDIT3) is implicated in apoptosis and differentiation and inhibits osteoclast differentiation via modulating the C/EBPα-CTSK axis [184]. DDIT3 expression was reduced in bone marrow-derived macrophages (BMM) during osteoclast development triggered by RANKL. The absence of DDIT3 elevates the expression of osteoclast-specific markers such as NFATc1, TRAP, CTSK, and DC- STAMP, as well as the number of actin loops [184]. Ensuring normal DDIT3 function is crucial for maintaining the bone remodeling process, and promoting DDIT3 is a significant aspect of treating bone loss.

**Figure 4:**
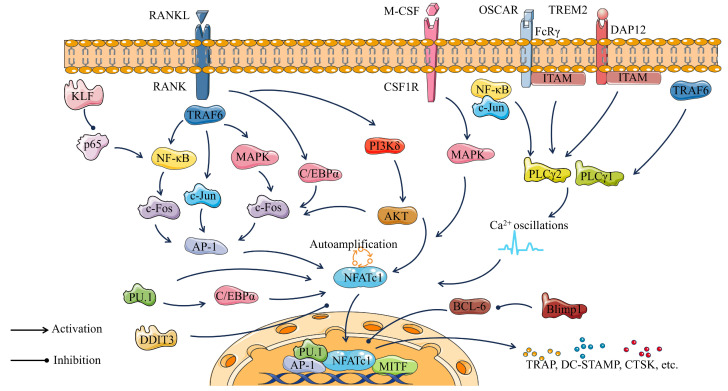
Osteoclastogenic pathway consisting of major transcription factors of osteoclasts.

Overall, osteoclast transcription factors play a crucial role in osteoclastogenesis. At the same time, osteoclast transcription factors serve as key nodes that constitute the pathways regulating osteoclastogenesis (Figure 4). Binding of RANKL to its receptor RANK activates C/EBPα, in addition to activating NF-κB, c-Jun, and MAPK through the recruitment of TRAF6. Activated c-Jun and c-Fos further promote AP-1 expression and ultimately NFATc1 expression. PU.1 promotes the expression of C/EBPα and also directly activates NFATc1. KLF2 plays an osteoclast inhibitory role related to the activation of NF-κB transcription through the control of p65. M-CSF and its receptor CSF-1R promote osteoclastogenesis by activating the MAPK cascade. PLCγ1 and PLCγ2 play important roles in osteoclast differentiation mainly by regulating DAG-mediated calcium oscillations and up-regulation of the transcription factor NFATc1. FcRγ, DAP12, and their associated partners (OSCAR and TREM2, respectively) promote osteoclast differentiation by inducing intracellular calcium (Ca^2+^) mobilization through activation of PLCγ2. In the nucleus, NFATc1 cooperates with other transcription factors, such as AP-1, PU.1, and MITF to induce various osteoclast-specific genes. Bcl6 can inhibit NFATc1 expression, and this inhibition is negatively regulated by Blimp1. Each of these transcription factors has the potential to be a key in the regulation of bone homeostasis.

## 6. Epigenetic Regulation of Transcription Factors in Osteoclastogenesis

Epigenetics refers to the study of mechanisms that can affect gene expression in a stable and potentially genetic manner without changing the DNA sequence. It describes three main phenomena: non-coding RNA sequences, methylated DNA modifications, and histone side chain modifications [185]. Epigenetic regulation of osteoclastogenesis plays an important role, and the exertion of this role is closely related to the regulation of osteoclast transcription factor expression.

### 6.1. Non-Coding RNAs (ncRNAs))

In both healthy and pathological contexts, non-coding RNAs (ncRNAs) fine-tuning gene expression programs are involved in bone homeostasis. Non-coding RNAs in osteoclast differentiation mainly include micro RNA (miRNA), long-non-coding RNA (lncRNA), and circular RNA (circRNA). Although each class of ncRNAs functions in a unique way, there are emerging connections between their methods for controlling gene expression. On the basis of newly acquired knowledge on the epigenetic regulation of gene expression for skeletal ncRNA regulation, new paths for translating miRNAs, lncRNAs, and cricRNAs into therapeutic targets for skeletal illnesses may be possible.

#### 6.1.1. MiRNA

Due to its capacity to regulate gene expression and provide post-transcriptional epigenetic modifications, miRNA has recently attracted attention. The class of non-coding RNAs known as miRNAs makes up only 1-5% of our genome and ranges in length from 18 to 25 nucleotides [77,186]. It is believed that miRNAs control up to 60% of human protein-coding genes, which are involved in many crucial life processes and may be controlled by binding to 30 untranslated regions (UTRs) [187,188].

The role of miRNAs in osteoclastogenesis includes both inhibition and promotion, involving the expression of multiple pathways and genes (Table 2). MiRNA-21 (miR-21) controls osteoclastogenesis through a number of intricate mechanisms. Estrogen inhibition of miR-21 expression causes an increase in Fas Ligand (FasL), which is a target of miR-21 in osteoclasts and induces autocrine osteoclast death [189]. RANKL induces miR-21 expression, which promotes osteoclastogenesis by down-regulating programmed cell death 4 (PDCD4) and subsequently regulating the c-Fos/NFATc1 axis [190,191]. In addition, miR-21 can regulate the balance of the RANKL/OPG ratio by inducing RANKL secretion from activated T cells and regulating OPG expression, which in turn affects the differentiation of osteoclasts [192]. However, miR-21-5p can inhibit osteoclast differentiation by acting on its target gene SKP2, as well as promote osteoclast apoptosis by directly interacting with the lncRNA GAS5 [193,194]. MiR-206-3p enhances trabecular bone architecture and reduces the number of osteoclasts by directly targeting bone morphogenetic protein-3 (BMP3) and NFATc1 to increase bone mass in Wnt1-cre; Gata4^fl/fl^ mice [195]. MiR-31 is induced by RANKL to optimize actin ring formation in osteoclasts by targeting RhoA expression, which is essential for cytoskeleton organization and bone resorption [196]. The most prevalent miRNA among those delivered is miR-92a-1-5p, who can down-regulate type I collagen expression by specifically targeting COL1A1, fostering osteoclast differentiation, and repressing osteoblastogenesis [197]. The exosome miR-1260b can inhibit its osteoclastogenic activity by targeting the Wnt5a-mediated RANKL pathway [198]. Several miRNAs are involved in regulating bone remodeling by inhibiting osteoclastogenesis. For instance, miR-125a-5p overexpression hinders the expression of TNFRSF1B and promotes osteoclast differentiation, while MiR-503-3p regulates osteoclast differentiation by decreasing Hpse gene expression [199,200]. MiRNA-based gene therapy is a rapidly evolving disease treatment strategy that has great potential for the treatment of bone-related diseases but faces many challenges due to the diversity of miRNA species and functions.

#### 6.1.2. LncRNA

LncRNAs are large non-coding RNAs with transcripts longer than 200 nt. LncRNAs play a crucial role in various activities of life by interacting with proteins, RNA, and DNA to regulate gene expression [212,213]. Their subcellular localization and functions are closely related. LncRNAs control gene expression at the transcriptional, post-transcriptional, and translational levels in the cytoplasm and at the epigenetic, transcriptional, and translational levels in the nucleus [213]. LncRNAs also play an important regulatory role in osteoclastogenesis (Table 3).

During osteoclast differentiation, LncRNA TUG1 is excessively expressed and controls the protein levels of anti-tartrate phosphatase (TRAP), NFATc1, and osteoclast-associated receptor (OSCAR). Meanwhile, it targets increased protein levels of V-maf muscular neurofibrosarcoma homolog B (Mafb) to promote osteoclast differentiation positively [214]. Lnc-AK077216 regulates NFATc1 expression, and the up-regulation or down-regulation of NFATc1 can promote or suppress osteoclast differentiation, bone resorption, and related gene expression, respectively [215]. Exosome lncRNA-SOX2OT is produced by non-small-cell lung cancer cells and targets the miRNA-194-5p/RAC1 signaling axis as well as the TGF-/pTHrP/RANKL signaling pathway in osteoclasts to regulate osteoclast differentiation and promote bone metastasis [216]. However, lncRNA CASC11, lncRNA CRNDE, lncRNA NEF, lncRNA SNHG1, and lncRNA XIXT have been shown to be up-regulated in postmenopausal osteoporosis [217,218,219,220,221]. During RANKL-induced osteoclast differentiation, the expression of lncRNA Bmncr was also reduced [222].

Although research on the regulatory mechanisms of lncRNA in osteoclast differentiation is still lacking, it has been shown that these molecules can serve as markers for diseases related to the bones and as therapeutic tools.

**Table 3:** LncRNAs in osteoclastogenesis.

LncRNAs	Roles	Target Gene(s)	References
LncRNA TUG1	Promoting osteoclast differentiation	TRAP, NFATc1, Mafb	[214]
LncRNA-AK077216	Bidirectional regulation of osteoclast differentiation	NFATc1	[215]
lncRNA Bmncr	Inhibiting osteoclast differentiation	RANKL	[222]
LncRNA-Jak3	Promoting osteoclast differentiation	NFATc1, CTSK	[223]
LncRNA XIST	Promoting osteoclast differentiation	SPHK1, S1P, ERK	[224]
LncRNA Nron	Inhibiting osteoclast differentiation	NF-κb, NFATc1	[225]
lncRNA-MIRG	Promoting osteoclast differentiation and bone resorption function	miR-1897, NFATc1	[226]
lncRNA NEAT1	Promoting osteoclast differentiation	M-CSF	[227]
lncRNA HOTAIR	Promoting osteoclast differentiation	RANKL	[228]
lncRNA SNHG15	Promoting osteoclast differentiation, proliferation, and metastasis	miR-381-3p/ NEK2	[229]

#### 6.1.3. CircRNA

CircRNAs are a class of RNA molecules with a stable ring-like structure discovered more than 40 years ago, which are produced by reverse splicing of linear RNAs covalently linked [230,231]. CircRNAs contain a large number of miRNA binding sites. They can contribute to bone remodeling by actively participating in bone-related signaling pathways, and intervening with the formation of the circRNA–miRNA–mRNA axis [186,232].

Except for a few CircRNAs that can regulate osteoclast transcription factors, the rest mostly play a role in regulating osteoclasteogenesis by regulating MiRNAs (Table 4). CircCHEK1_246aa, a cyclic CHEK1 RNA that encodes and is translated into the catalytic center of CHEK1 kinase, can promote osteoclast formation by affecting NFATc1 expression [233]. CircRNA 28313 is significantly induced by RANKL and colony-stimulating factor 1 (CSF1) co-treatment and alleviates miR-195a-mediated inhibition of CSF1 by acting as a ceRNA, thereby regulating osteoclast differentiation [234]. Similarly, by acting as a ceRNA for miR-5107, circRNA 009934 promotes osteoclastogenesis and regulates TRAF6 expression [235]. The RNA methylation enzyme METTL3 can promote competitive binding of circ_0008542 to miRNA-185-5p by acting on the 1956bp m6A functional site in circ_0008542, and ultimately lead to an increase in the target gene RANK and the initiation of osteoclast bone resorption [236]. Circ_0007059 regulates osteoclastogenesis via the miR-378/bone morphogenetic protein 2 (BMP-2) signaling pathway [237]. In addition, circHmbox1 inhibits RANKL-induced osteoclast differentiation, particularly by binding to miR-1247-5p [79].

In general, CircRNAs are frequently combined with miRNAs to regulate the differentiation of osteoclasts. The combination of circRNAs with miRNAs shows significant potential for treating bone-related diseases.

**Table 4:** CircRNAs in osteoclastogenesis.

CircRNAs	Roles	Target Gene(s)	References
CircCHEK1_246aa	Promoting osteoclast differentiation	NFATc1	[233]
CircRNA 28313	Promoting osteoclast differentiation	CSF1, miR-195a	[234]
CircRNA 009934	Promoting osteoclast differentiation	miR-5107, TRAF6	[235]
Circ_0008542	Promoting osteoclast differentiation and bone resorption function	METTL3, miRNA-185-5p	[236]
Circ_0007059	Inhibiting osteoclast differentiation	miR-378, BMP-2	[237]
CircHmbox1	Inhibiting osteoclast differentiation and promoting osteoblast differentiation	miRNA-1247-5p	[79]

### 6.2. DNA and Histone Modifications

DNA methylation, as an epigenetic event, plays a key role in the pathogenesis, progression, and treatment of human diseases, as well as in the osteoclast differentiation process [238,239]. In addition, DNA methylation may mediate the “reprogramming” of monocytes in vivo, allowing them to “remember” age, menopausal state, and bone formation state in vitro, and result in more destructive osteoclasts [240]. It has also been revealed that PU.1, NF-κB, and AP-1 (Jun/Fos) are involved in DNA methylation changes, of which only PU.1 is involved in both hypermethylation and hypomethylation [148]. PU.1 can be involved in driving hypermethylation and hydroxymethylation-mediated hypomethylation by interacting with DNMT3b and TET2, and knockdown of PU.1 impairs the access to DNA methylation and expression changes [148].

DNA of each cell is wrapped around histone octamers that form what are known as “nucleosomal core particles” [241]. The tails of these histones protrude from the nucleosomal, and many residues in these tails can be post-translationally modified to affect all DNA-based processes, including gene expression; DNA replication, repair, and stemness; chromosome cohesion and segregation; and changes in cellular state [241,242]. Significantly, histone acetyltransferase (HAT) and histone deacetylase (HDAC) are crucial to this process [243].

RANKL increases NFATc1 transcriptional activity during subsequent osteoclast differentiation by inducing HAT-mediated NFATc1 acetylation and stabilization [244]. HDAC1 is expressed in osteoclast precursors and then markedly decreased after RANKL stimulation. Recruitment to osteoclast gene promoters, including NFATc1 and Oscar, can hinder osteoclastogenesis by blocking the expression of the genes [245,246]. In addition, HDAC7, HDAC9, HDAC10, Sirtuin 1, Sirtuin 3, Sirtuin 6, etc., also inhibit osteoclastogenesis. HDAC3, expressed in osteoclast precursors, maintains a low expression during osteoclast differentiation but has a positive effect on the osteoclast genes NFATc1, Ctsk, and Dc-stamp and on the formation of the actin ring [61,246]. HDAC11 plays a role in osteoclast fusion as well as activity by increasing Dc-stamp and Ctsk expression, but it is not associated with regulation of Nfatc1 expression or activity [247]. The role of HDACs in osteoclastogenesis has been studied with some success, but there are still many mechanisms that have not been elucidated, such as HDAC11, HDAC10, HDAC8, HDAC4, HDAC5, and so on.

### 6.3. Modulating Epigenetic Regulators as Therapy for Bone Disorders

It could be a novel strategy to treat bone-related disorders by using epigenetic regulators to modify osteoclast gene expression, therefore regulating osteoclast differentiation, function, and apoptosis. Proteolysis of the histone H3 N-terminal tail (H3NT) via matrix metalloproteinase-9 (MMP-9) is a critical mechanism for activating gene expression during osteoclast differentiation. The activity of MMP-9 on H3NT is tightly controlled by histone modifications, including H3K18 acetylation (H3K18ac) and H3K27 monomethylation (H3K27me1). These modifications are essential for the stable interaction of MMP-9 with nucleosomes during H3NT proteolysis [248]. Treatment of RANKL-induced osteoclast progenitor cells (OCP) with the DNMT inhibitor 5-Aza-2′-deoxycytidine (5-Aza-CdR) induced CpG island hypomethylation and promoted MMP-9 transcription, which in turn led to a marked enhancement of H3NT proteolysis and OCP cell differentiation [236]. However, treatment of OCP-induced cells with the HDAC inhibitor trichostatin A (TSA) stimulated H3K27ac, but was accompanied by a decrease in H3K27me1, ultimately leading to impaired osteoclastogenic gene expression [248].

A novel DNA methyltransferase 3a (Dnmt3a)-mediated S-adenosylmethionine (SAM)-mediated DNA methylation regulates osteoclastogenesis through epigenetic suppression of antiosteoclastogenic genes, the defect of which leads to inhibition of osteoclast differentiation [249]. In addition, theaflavin-3,3′-digallate can eliminate bone loss in osteoporosis models by inhibiting DNA methylation [249].

Epigenetic studies have provided new strategies for regulating osteoclastogenesis, which is inextricably linked to transcription factors such as NFATc1. Nevertheless, further comprehensive and systematic research is essential to investigate the epigenetic regulation of osteoclastogenesis more profoundly, and reliable approaches to control transcription factors connected with osteogenesis remain to be deeply studied.

## 7. Conclusions

Osteoclasts are key cells with a bone resorption function, derived from HSCs and EMPs, and are essential for bone remodeling and bone homeostasis. The process of osteoclastogenesis is tightly regulated by several transcription factors, of which NFATc1 is key. The expression of transcription factors in osteoclasts can be regulated through three major aspects of epigenetics: methylated DNA modifications, histone side chain modifications, and non-coding RNA sequences. Intercellular communication leads to changes in the micro-environment, during which substances such as cytokines are produced that can have a major impact on cell differentiation, function, and survival. Communication between osteoclasts and related cells, mainly osteoblasts, osteocytes, immune cells, and adipocytes, also exerts a regulatory effect on transcription factors of osteoclasts. The mechanisms of action of transcription, as well as the targets and pathways that regulate transcription factor expression, addressed in this review may become important directions for the treatment of bone-related diseases in the future.

## Figures and Tables

**Table 1:** Drugs that act on osteoclasts to treat bone-related diseases clinically.

	Drug Names	Functions	Side Effects	References
**Anti-resorptive**				
RANKL antibody	Denosumab (Prolia)	Inhibiting the formation, function, and survival of osteoclasts	Rebound vertebral fractures	[12,13]
Selective Estrogen Receptor Modulators (SERMs)	Raloxifene (Evista)	Regulating the survival of mature osteoclasts	Venous thromboembolism and stroke	[13,18]
Cathepsin K Inhibitors	Odanacatib (III clinical trials)	Inhibiting the late differentiation of osteoclasts	Stroke	[14]
Calcitonin	Calcitonin (Miacalcin)	Blocking the maturation of osteoclast precursors and regulating the function of osteoclasts	Rhinitis, nasal irritation, back pain, nosebleed, and headache	[13,19]
Bisphosphonates (BPs)	Alendronate (Binosto, Fosamax), risedronate (Actonel, Atelvia), ibandronate (Boniva, Bondronat)	Induction of apoptosis in osteoclasts	Atypical femoral fractures, osteonecrosis of the jaw, gastrointestinal and renal complications, osteomalacia	[20,21,22]
**Anabolic**				
Strontium	Strontium ranelate (Protelos)	Interfering with osteoclast differentiation and promoting osteoclast differentiation	Heart problems	[23]

**Table 2:** MiRNAs in Osteoclastogenesis.

MiRNAs	Roles	Target Gene(s)	References
MiR-21	Inhibiting apoptosis of osteoclasts Promoting osteoclast differentiation	FasL PDCD4	[189,190,191]
MiR-21-5p	Inhibiting osteoclast differentiation Promoting apoptosis of osteoclasts	SKP2	[193,194]
MiR-31	Promoting osteoclast skeleton formation	RhoA	[196]
MiR-92a-1-5p	Promoting osteoclast differentiation and repressing osteoblastogenesis	COL1A1	[197]
MiR-1260b	Inhibiting osteoclast differentiation	Wnt5a	[198]
MiR-503-3p	Inhibiting osteoclast differentiation	Hpse	[200]
MiR-182	Promoting osteoclast differentiation	RBP-J and NFATc1	[201]
MiR-199a-5p	Promoting osteoclast differentiation	Mafb	[202]
MiR-506-3p	Inhibiting osteoclast differentiation and the release of bone-resorbing enzymes	RANKL and NFATc1	[203]
MiR-206-3p	Inhibiting osteoclast differentiation	BMP3 and NFATc1	[195]
MiR-30a	Inhibiting osteoclast differentiation and skeleton formation	DC-STAMP, c-Fos, NFATc1	[204]
MiR-483-5p	Promoting osteoclast differentiation	NFATc1, NFAT2, CTSK	[205]
MiR-133a	Promoting osteoclast differentiation	NFATc1, c-Fos, TRAP	[206]
MiR-124	Inhibiting osteoclast differentiation and promoting osteoclast differentiation	NFATc1 Pten/PI3K/Akt	[207,208]
MiR-340	Inhibiting osteoclast differentiation	MITF	[209]
MiR-1276	Inhibiting osteoclast differentiation	MITF	[138]
MiR-214-5p	Promoting osteoclast differentiation	ITGA7	[210]
MiR-7b	Inhibiting osteoclast differentiation	DC-STAMP	[211]
